# Prevalence and Molecular Characterization of Methicillin-Resistant Staphylococci Recovered from Public Shared Bicycles in China

**DOI:** 10.3390/ijerph19084492

**Published:** 2022-04-08

**Authors:** Zhen Xu, Liqin Chen, Xiaowei Chen, Amei Tang, Dengmin Huang, Qin Pan, Zhongze Fang

**Affiliations:** 1 Department of Toxicology and Sanitary Chemistry, School of Public Health, Tianjin Medical University, Qixiang Road No. 22, Tianjin 300070, China; chenliqin@tmu.edu.cn (L.C.); 15822330801@163.com (X.C.); t159357am@163.com (A.T.); huangmin5433@163.com (D.H.); lixia20211224@163.com (Q.P.); 2 Tianjin Key Laboratory of Environment, Nutrition and Public Health, Tianjin Medical University, Qixiang Road No. 22, Tianjin 300070, China; 3 Center for International Collaborative Research on Environment, Nutrition and Public Health, School of Public Health, Tianjin Medical University, Qixiang Road No. 22, Tianjin 300070, China

**Keywords:** public shared bicycle, staphylococci, antimicrobial resistance, *mecA*, SCC*mec*, MLST

## Abstract

Millions of public shared bicycles (PSBs) have been launched in China, and PSBs are a potential reservoir of antimicrobial-resistant staphylococci. However, no national data to elucidate the dissemination, antimicrobial resistance and genotypes of staphylococci has been recovered from public shared bicycles located in different cities in China. Antimicrobial susceptibility, SCC*mec* types and sequence types of staphylococci were determined. A total of 146 staphylococci were recovered in this study, and 87% staphylococcal isolates were resistant to at least one antibiotic. In total, 29 (20%) staphylococcal isolates harbored *mecA* gene, and SCC*mec* types were determined as follows: SCC*mec* type II (*n* = 1), IV(*n* = 3), V (*n* = 4), VI (*n* = 1), VIII (*n* = 2), A/1 (*n* = 6), A/5 (*n* = 2), C/1 (*n* = 2), C/2 (*n* = 1), C/3 (*n* = 1), (*n* = 5) and Pseudo (ψ)-SCC*mec* (*n* = 1). Sequence types of 16 *Staphylococcus epidermidis* were determined, including ST10, ST17, ST59, ST60, ST65, ST130, ST184, ST262, ST283, ST337, ST360, ST454, ST567, ST820, ST878 and ST934. PSBs are a reservoir of diverse antimicrobial-resistant staphylococci, and staphylococcal species differences were observed in isolates that were recovered from public shared bicycles in the south and north of China. PSBs are a source of antimicrobial resistance and genetic diverse staphylococci.

## 1. Introduction

The GPS and smartphone-based bicycle rental system was implemented in China in 2016 [1], and since then millions of shared bicycles have been launched all over the country [2]. Citizens have benefited from the convenience of using public shared bicycles, which solves the last mile traffic gap [3]. However, the issue of hygiene has been raised in relation to using public shared bicycles [2].

Xu et al. were the first to report the dissemination of antimicrobial-resistant staphylococci that were recovered from public shared bicycles in Tianjin, China [2], and then Zou et al. stated the prevalence of antimicrobial-resistant *Enterobacteriaceae* that were isolated from shared bikes in Beijing, China [4]. Three recent studies by Wu and co-workers demonstrated the diversity and transmission risks of antimicrobial-resistant bacteria in Chengdu, China [1,3,5].

Staphylococci and enterococci were used to study the role of public shared bicycles in circulating the antimicrobial resistance between hospitals, metro-stations, schools and riders [3]. Coagulase-negative staphylococci (CoNS) are a commensal bacteria of human and animal skin [6], and are becoming more and more known as a significant cause of human and animal infections [7]. Moreover, it is known that staphylococci were the most abundant bacteria that were recovered from frequently touched surfaces [1,8]. *Staphylococcus epidermidis* (*S. epidermidis*) is one of the major species of CoNS, but also an important opportunistic pathogen causing a wide range of infections [9]. Epidemic clonal lineages of hospital associated methicillin-resistant *S. epidermidis* (MRSE) have been well documented [10]. However, the environmental clonal lineages of MRSE clones, especially the clonal lineages of MRSE that were recovered from public shared bicycles in mainland China, remains unknown.

Millions of public shared bicycles have been put into use all over the country, however, the dissemination and antimicrobial resistance of human-associated bacteria that were recovered from public shared bicycles were available in three cities (Beijing, Tianjin and Chengdu) in China [1,2,3,4,5], which is insufficient to represent the issue in other parts of China. Therefore, the *Staphylococcaceae*, which are commonly associated with public shared bicycles, were selected for a surveillance study to address dissemination, antimicrobial resistance and molecular characterization of staphylococci that were isolated from public shared bicycle in China.

## 2. Materials and Methods

Sterilized cotton swabs (Transystem™ 108C, COPAN, Corona, CA, USA) were used to collect public shared bicycles samples. The procedure was as follows: sterilized cotton swabs were smeared on the handrails of public shared bicycles several times, and the collection area is 5 cm × 5 cm of both handrails. The swabs were then stored in culture medium and transferred back into the laboratory within 24 h. The collection personnel were trained before collection.

Swabs were then plated on nutrient agar plates and cultured at 37 °C for 24 h. The colonies of each plate were differentiated by shape and color, and only one colony was inoculated when there are several colonies with same shape and color.

All purified isolates were identified by Matrix-assisted laser desorption ionization time-of-flight mass-spectroscopy (MALDI-TOF-MS, Microflex LT, Bruker Daltonics, Coventry, UK) The sample preparation were followed the manufacturers’ guidance. Briefly, 1 loop of fresh culture was mixed with 300 μL double distilled water, and then mixed with 900 μL ethanol. The membrane protein was extracted by formic acid and acetonitrile, and 1 μL extracts were plated on the target plate. Afterwards 1 μL OS solution was then overlaid on the dried extracts. The target plate was then analyzed by MALDI-TOF MS in a positive linear mode (2000–20,000 m/z range). Then the resulting spectra were compared with reference spectra by using the Biotyper 3.0 software (Bruker Daltonics, Coventry, UK). *Escherichia. coli* DH5α (Bruker Daltonics, Coventry, UK) was used as a standard for calibration and quality control [11].

Antimicrobial susceptibility of staphylococci was determined by disc-diffusion methods, and 11 antibiotic discs were used, including: chloramphenicol (C, 30 µg), clindamycin (CD, 2 μg), gentamicin (CN,10 µg), ceftaroline (CPT,30 µg), erythromycin (E, 15 µg), cefoxitin (FOX, 30 µg), levofloxacin (LEV, 5 µg), linezolid (LZD, 30 µg), penicillin (PG,10 unit), tetracycline (T, 30 µg), teicoplanin (TEC, 30 µg) (MAST Group Ltd., Reinfeld, Germany). The resistance, susceptibility and intermediate resistance were interpreted by CLSI Performance standard of Antimicrobial Susceptibility Testing (CLSI: 27th edition) [12].

*mecA* gene was detected by using specific primers: mA1 (5′-TGCTATCCACCCTCAAACAGG-3′) and mA2 (5′-AACGTTGTAACCACCCCAA GA-3′) that were described by Kondo et al. [13]. The SCC*mec* types were assigned by the combination of *mec* gene complex and *ccr* gene complex. The *mec* gene complex and *ccr* gene complex were amplified by the procedure that were described by Kondo et al. [13].

Sequence type (ST) of each *S. epidermidis* were determined by amplification and sequencing of seven housekeeping genes. The primers and PCR procedure were described on the website of Public databases for molecular typing and microbial genome diversity. The sequence type (ST) were then assigned by Public databases for molecular typing and microbial genome diversity (https://pubmlst.org, accessed on 5 April 2021).

## 3. Results

### 3.1. Identification of Isolates

In total, 222 swabs were collected from 12 cities that belonged to 11 provinces and the capital city of China: Beijing, Chengdu, Kunming, Lanxi, Putian, Puyang, Shihezi, Suzhou, Shuozhou, Xining, Yinchuan and Zhuzhou. A total of 146 staphylococcal isolates that belonged to 13 species were recovered (Table 1).

The 13 staphylococcal species were determined as follows: *Staphylococcus arlettae* (*n =* 2, *S. arlettae*), *Staphylococcus capitis* (*n =* 3, *S. capitis*), *Staphylococcus epidermidis* (*n =* 16, *S. epidermidis*), *Staphylococcus gallinarum* (*n =* 1, *S. gallinarum*), *Staphylococcus haemolyticus* (*n =* 4, *S. haemolyticus*), *Staphylococcus hominis* (*n =* 95, *S. hominis*), *Staphylococcus kloosii* (*n =* 1, *S. kloosii*), *Staphylococcus pseudintermedius* (*n =* 1, *S. pseudintermedius*), *Staphylococcus saprophyticus* (*n =* 3, *S. saprophyticus*), *Staphylococcus sciuri* (*n =* 1, *S. sciuri*), *Staphylococcus succinus* (*n =* 10, *S. succinus*), *Staphylococcus warneri* (*n =* 2, *S. warneri*) and *Staphylococcus xylosus* (*n =* 7, *S. xylosus*) (Table 2 and Table S1).

### 3.2. Antimicrobial Susceptibility Profile of Staphylococcal Isolates

Antimicrobial susceptibility tests were applied to all staphylococcal isolates. In total, 75 (51%) staphylococcal isolates were resistant to penicillin, followed by 54 (37%) that were resistant to erythromycin, 41 (28%) to tetracycline, 29 (20%) to cefoxitin, 20 (14%) to clindamycin, 17 (12%) to linzolid, 14 (10%) to teicoplanin, 7 (5%) to chloramphenicol, 3 (2%) to gentamicin, 2 (1%) to levofloxacin and 2 (1%) to ceftaroline (Table 2 and Table S1).

### 3.3. mecA Gene Detection and SCCmec Typing of Staphylococcal Isolates

All staphylococcal isolates were tested for carriage of the *mecA* gene. It is found that the *mecA* gene was carried by 29 staphylococcal isolates, including 6 *S. epidermidis*, 3 *S. haemolyticus*, 18 *S. hominis*, 1 *S. warneri* and 1 *S. xylosus*. One isolate carried SCC*mec* type II, three isolates harboured SCC*mec* type IV, four isolates carried SCC*mec* type V, one SCC*mec* type VI and two SCC*mec* type VIII. In addition, six isolates carried SCC*mec* A/1, two isolates SCC*mec* A/5, two isolates SCC*mec* C/1, one SCC*mec* C/2 and one SCC*mec* C/3. SCC*mec* types of six isolates cannot be assigned due to the lack of either the *mec* gene complex or the *ccr* gene complex, which were named as SCC (*n =* 5) and Pseudo (ψ)-SCC*mec* (*n =* 1) (Table 2).

### 3.4. Multi-Locus Sequence Typing of S. epidermidis

Multi-locus sequence types were determined for all 16 *S. epidermidis* isolates, and none of them shared the same sequence type (ST). The 16 STs were as follows: ST10, ST17, ST59, ST60, ST65, ST130, ST184, ST262, ST283, ST337, ST360, ST454, ST567, ST820, ST878 and ST934 (Table 2).

## 4. Discussion

In this study, we had an insight into the dissemination, antimicrobial resistance and molecular characterization of staphylococci that were recovered from PSB in 12 cities in China.

Zou et al., Xu et al. and Gu et al. found that *Staphylococcus* and *Enterococcus* were widely disseminated on PSBs in Beijing, Chengdu and Tianjin [2,3,4]. Consistent with their report, *Staphylococcaceae* were recovered from the PSBs of 12 cities in this study. In addition, Gu et al. found that *S. epidermidis* (12.9%) was the most prevalent species that was recovered from PSBs, followed by *S. sciuri*, *S. haemolyticus*, *S. aureus* and *S. hominis* [3]. In contrast with Gu’s study, we found that *S. hominis* was the most prevalent species. Moreover, *S. arlettae*, *S. gallinarum*, *S. kloosii*, *S. pseudintermedius*, *S. saprophyticus*, *S. succinus*, *S. warneri* and *S. xylosus* that were isolated in this study have not been reported to be recovered from PSB samples before [2]. No *S. aureus* were recovered in this study. PSBs were significantly affected by human microbiota in China, which may indicate insufficient bike cleaning.

*Staphylococcus* were recovered from PSBs in both the south and north of China (Table 1). Moreover, some staphylococcal species, such as *S. saprophyticus* and *S. succinus*, were isolated from PSB samples that were collected from samples of cities in the north of China. *S. saprophyticus* is the second most common cause of urinary tract infection [14], and *S. succinus* is commonly found in Bovine mastitis [15]. The dissemination of *S. saprophyticus* and *S. succinus* may pose a public health threat to the vulnerable population. *S. arlettae*, *S. capitis*, *S. gallinarum* and *S. kloosii* were specifically found in PSB samples that were collected from samples of south cities of China (Table 1). Recently, *S. capitis* has been reported to be the cause of endogenous endophthalmitis of a 34-year-old male patient [16]. The pathogenesis and virulence of environmental CoNS warrant much attention.

The staphylococcal antimicrobial susceptibility rate towards penicillin, erythromycin, tetracycline, cefoxitin, clindamycin, and linezolid of this study were generally lower than the rates that were reported in Chengdu [3], which suggested the varied antimicrobial susceptibility rate of staphylococci that were recovered from PSB samples in different cities of China. In this study, 70% of the isolates that were recovered from PSB samples in Chengdu were resistant to penicillin, which was consistent with Gu et al.’s report [3]. Moreover, 88% of the isolates that were recovered from Xining PSB samples showed the highest resistant rates towards penicillin, while staphylococcal isolates that were recovered from PSB samples of Beijing have the lowest resistant rate (20%). Moreover, the average resistant rate of staphylococcal isolates towards cefoxitin were 20% in this study, which was lower than the rates that were reported in Chengdu (63.1%) [3]. Multiple-resistant staphylococci were widely disseminated in cities of China.

*mecA* gene confers methicillin resistance of staphylococci [17], and 29 *mecA*-gene positive isolates were recovered from PSB samples that were collected from 8 (8/12, 67%) cities (Beijing, Kunming, Putian, Puyang, Shuozhou, Suzhou, Shihezi and Xining). The majority of *mecA*-gene positive isolates (41%) were recovered from Suzhou, south part of China. A total of 29 (20%) staphylococci were *mecA* positive, which was lower than the rate that was reported in Tianjin [2].

SCC*mec* types I, II, III are reported to be associated with MRSA isolated from healthcare settings, whereas SCC*mec* IV and V are mainly associated with community setting [18]. In this study, 24% of staphylococcal isolates carried community-associated SCC*mec*, and one isolate harboured hospital-associated SCC*mec* types. SCC*mec* type VI is different from SCC*mec* type IV because it contains a new *ccrAB* allotype, and SCC*mec* IV and VI were both firstly identified in a pediatric clone (ST5) of *S. aureus* [19,20]. In this study, SCC*mec* type VI was identified in *S. epidermidis*. SCC*mec* VIII was first reported in 2009, which was recovered from a patient in Canada [16]. This was the first time that SCC*mec* type VIII element has been identified from the environment in China. Bouchami et al. reported that the *mec* gene complex A and the type 1 *ccr* complex are prevalent in *S. hominis* [21], and our results were consistent with his findings. It is worth noting that Pseudo (ψ)-SCC*mec* is defined as processing the *mec* complex but lacks *ccr* [9]. ψ SCC element is characterized by lacking genes for *ccr* and *mec* [9]. In this study, six isolates could not be assigned due to lack of the *mec* or *ccr* complex. Unassigned SCC*mec* elements were widely found in staphylococci that were recovered from PSBs in China.

In total, 16 *S. epidermidis* belonged to 16 different ST, which indicated the genetic intricacy of *S. epidermidis* that were recovered from PSBs in China. *S. epidermidis* ST59 is among the community-associated genotypes, which is responsible for various infections in Asia [6]. Moreover, ST 17 [22] and 130 [23] were previously recovered from clinical samples, and ST60 was reported to be isolated from diseased cats and dogs [24]. *S. epidermidis* ST10 was reported to be the cause of implant associated infections [25], and one such clone was identified on PSBs in this study. The dissemination of pathogenic clones on PSBs might pose a health threat to the public. In addition, ST65, 184, 262, 283, 337, 360, 454, 567, 820, 878 and 934 are rarely reported. PSBs may act as a reservoir of *S. epidermidis* with diverse genotypes. Pathogenic clones that were recovered from PSBs are a concerning finding.

## 5. Conclusions

In conclusion, *Staphylococcus* were commonly found on PSBs in China. Staphylococcal species differences were observed between isolates that were recovered from PSBs in the south and north of China. The antimicrobial susceptibility rates of staphylococcal isolates varied between cities, and *mecA* gene-positive isolates were recovered from PSB samples of 8 cities (66%). Diverse SCC*mec* elements were found in staphylococcal isolates, and genotypic diversity was observed in *S. epidermidis*. The bacterial species, antimicrobial resistance ratio, carriage rate of *mecA* gene and genotypes were different between cities, which suggested varying hygiene levels. Therefore, a local monitoring study should be implemented from time to time, so as to notify the local government to take cleaning strategies according to the local circumstances.

## Figures and Tables

**Table 1:** Staphylococcal species that were recovered from cities in the south and north of China.

Genus	Species Recovered from PSBs Samples Collected from 12 Cities of China		
	All Cities ^a^	Northern Cities ^b^	Southern Cities ^c^
*Staphylococcus*	*S. epidermidis*, *S. haemolyticus*, *S. hominis*, *S. xylosus*	*S. saprophyticus*, *S. succinus*	*S. arlettae*, *S. capitis*, *S. gallinarum*, *S. kloosii*, *S. pseudintermedius*, *S. sciuri*, *S. warneri*

Note: ^a^—species that were recovered from PSB samples of southern and northern cities; ^b^—species that were recovered from PSB samples of northern cities (Beijing, Ningxia, Puyang, Shihezi, Shuozhou, Xining); ^c^—species that were recovered from PSB samples of Southern cities (Chengdu, Kunming, Lanxi, Putian, Suzhou, Zhuzhou).

**Table 2:** Antimicrobial susceptibility and molecular characterization of *S. epidermidis* *and mecA*-positive staphylococci recovered from PSBs.

No	ID	Sites	Bicycle Type	Cities	Species	CD	GM	E	PG	FOX	T	LZD	C	LEV	CPT	TEC	*mecA*	SCC*mec*	ST
1	SC3004	handrail	Hellobike	Chengdu	*S. epidermidis*	I	S	I	R	S	R	S	S	S	S	R	-	-	10
2	SC3003	handrail	Hellobike	Chengdu	*S. epidermidis*	I	S	R	R	S	R	R	R	I	R	R	-	-	17
3	SC0607	handrail	Hellobike	Chengdu	*S. epidermidis*	S	S	S	S	S	S	S	S	S	S	S	-	-	60
4	HZZ47	handrail	Zhuzhou bicycle	Zhuzhou	*S. epidermidis*	S	S	I	R	S	S	S	S	S	S	I	-	-	65
5	NX35	handrail	Mobike	Yinchuan	*S. epidermidis*	R	S	R	R	S	S	S	S	I	S	S	-	-	130
6	NX4203	handrail	Mobike	Yinchuan	*S. epidermidis*	S	S	S	S	S	S	S	S	S	S	S	-		337
7	HPY1904	handrail	Hellobike	Puyang	*S. epidermidis*	S	S	S	S	S	S	S	S	S	S	S	-	-	567
8	HPY1905	handrail	Hellobike	Puyang	*S. epidermidis*	I	S	S	R	S	S	S	S	S	S	S	-	-	283
9	HPY50	handrail	Hellobike	Puyang	*S. epidermidis*	S	S	S	S	S	S	S	S	S	S	S	-	-	820
10	JS3903	handrail	Suzhou bicycle	Suzhou	*S. epidermidis*	I	S	S	S	S	S	S	I	S	S	R	-	-	878
11	JS3902	handrail	Suzhou bicycle	Suzhou	*S. epidermidis*	S	S	S	R	R	S	S	S	S	S	S	+	C/3	184
12	YN4603	handrail	Mobike	Kunming	*S. epidermidis*	I	S	R	R	R	S	S	S	S	S	I	+	V	360
13	YN2601	handrail	Qingju bicycle	Kunming	*S. epidermidis*	I	S	I	R	R	R	S	S	I	S	I	+	V	934
14	JS5005	handrail	Suzhou bicycle	Suzhou	*S. epidermidis*	R	S	R	R	R	R	R	S	S	S	R	+	VI	262
15	HPY43	handrail	Hellobike	Puyang	*S. epidermidis*	R	S	R	R	R	R	S	S	I	S	S	+	VIII	59
16	JS0601	handrail	Suzhou bicycle	Suzhou	*S. epidermidis*	I	S	R	R	R	R	S	S	S	S	I	+	VIII	454
17	JS0602	handrail	Suzhou bicycle	Suzhou	*S. haemolyticus*	R	S	R	R	R	S	S	S	S	I	R	+	C/1	
18	JS0603	handrail	Suzhou bicycle	Suzhou	*S. haemolyticus*	R	S	S	R	R	S	S	S	S	R	S	+	C/2	
19	JS4103	handrail	Suzhou bicycle	Suzhou	*S. haemolyticus*	S	R	R	R	R	R	S	S	S	I	R	+	V	
20	BJ1103	handrail	Hello bicycle	Beijing	*S. hominis*	S	S	S	S	R	S	S	S	S	S	S	+	A/1	
21	JS4101	handrail	Suzhou bicycle	Suzhou	*S. hominis*	S	S	R	R	R	R	S	S	S	S	S	+	A/1	
22	JS4102	handrail	Suzhou bicycle	Suzhou	*S. hominis*	S	S	R	R	R	S	S	S	S	S	S	+	A/1	
23	SX3302	handrail	Hello bicycle	Shuozhou	*S. hominis*	S	S	S	S	R	S	S	S	S	S	S	+	A/1	
24	XJ2903	handrail	Shihezi bicycle	Shihezi	*S. hominis*	I	S	R	R	R	R	R	S	I	I	I	+	A/1	
25	XJ2904	handrail	Shihezi bicycle	Shihezi	*S. hominis*	I	S	R	R	R	R	S	S	S	S	S	+	A/1	
26	XJ2101	handrail	Shihezi bicycle	Shihezi	*S. hominis*	I	S	S	S	R	S	S	S	S	S	S	+	A/5	
27	XJ3502	handrail	Shihezi bicycle	Shihezi	*S. hominis*	I	S	S	S	R	S	S	S	S	S	S	+	A/5	
28	JS4202	handrail	Suzhou bicycle	Suzhou	*S. hominis*	S	S	R	R	R	S	S	S	S	S	S	+	C/1	
29	JS4104	handrail	Suzhou bicycle	Suzhou	*S. hominis*	R	S	R	R	R	S	S	S	S	S	S	+	IV	
30	QH07	handrail	Xining bicycle	Xining	*S. hominis*	S	S	S	R	R	S	S	S	S	S	S	+	IV	
31	YN39	handrail	Kunming bicycle	Kunming	*S. hominis*	S	S	S	S	R	S	S	S	I	S	S	+	II	
32	FJ45	handrail	Ubike	Putian	*S. hominis*	S	S	R	S	R	S	S	R	S	S	S	+	V	
33	JS07	handrail	Suzhou bicycle	Suzhou	*S. hominis*	R	S	R	R	R	S	S	S	S	S	I	+	SCC	
34	JS1003	handrail	Suzhou bicycle	Suzhou	*S. hominis*	S	S	S	R	R	S	S	S	S	S	S	+	Pseudo (ψ)-SCC*mec*	
35	XJ0102	handrail	Shihezi bicycle	Shihezi	*S. hominis*	S	S	R	R	R	S	S	S	S	S	S	+	SCC	
36	XJ3602	handrail	Shihezi bicycle	Shihezi	*S. hominis*	I	S	I	R	R	R	S	I	S	S	S	+	SCC	
37	XJ4803	handrail	Shihezi bicycle	Shihezi	*S. hominis*	I	S	R	S	R	S	S	S	S	S	S	+	SCC	
38	YN40	handrail	Mobike	Kunming	*S. warneri*	I	S	R	R	R	S	S	I	S	S	I	+	IV	
39	YN4602	handrail	Mobike	Kunming	*S. xylosus*	I	S	I	R	R	R	S	S	I	S	S	+	SCC	

Note: C—chloramphenicol (30 µg); CD—clindamycin (2 μg); CN—gentamicin (10 µg); CPT—ceftaroline (30 µg); E—erythromycin (15 µg); FOX—cefoxitin (30 µg); LEV—levofloxacin (5 µg); LZD—linezolid (30 µg); P—penicillin (10 unit); T—tetracycline (30 µg); TEC—teicoplanin (30 µg).

## Data Availability

The data presented in this study are available in this article and Supplementary Materials.

## Supplementary Materials

The following supporting information can be downloaded at: https://www.mdpi.com/article/10.3390/ijerph19084492/s1, Table S1: Antimicrobial susceptibility of mecA-negative staphylococci recovered from PSBs.
