# Supplementary material for: Prevalence and Molecular Characterization of Methicillin-Resistant Staphylococci Recovered from Public Shared Bicycles in China
Source: Int J Environ Res Public Health. 2022 Apr 8;19(8):4492. doi: 10.3390/ijerph19084492 (PMC9027712; doi:10.3390/ijerph19084492)
Supplement: Supplementary file 1 [file ijerph-19-04492-s001.zip › ijerph-1659642-supplementary.pdf]

Table S1. Antimicrobial susceptibility of mecA-negative staphylococci recovered from PSBs.

No	ID	sites	Type	cities	Species	CD	GM	E	PG	FOX	T	LZD	C	LEV	CPT	TEC
1	FJ0601	handrail	Ubike	Putian	*Staphylococcus arlettae*	R	S	R	R	S	S	S	I	S	S	I
2	fj0604	handrail	Ubike	Putian	*Staphylococcus arlettae*	I	S	R	S	S	S	S	S	S	S	S
3	JS2402	handrail	Suzhou bicycle	Suzhou	*Staphylococcus capitis*	S	S	I	R	S	S	S	S	S	S	S
4	JS2404	handrail	Suzhou bicycle	Suzhou	*Staphylococcus capitis*	I	S	R	R	S	S	S	S	S	S	I
5	YN45	handrail	Mobike	Kunming	*Staphylococcus capitis*	S	S	S	S	S	S	S	S	S	S	S
6	SC4102	handrail	Mobike	Chengdu	*Staphylococcus gallinarum*	I	S	I	R	S	S	R	I	S	I	R
7	XJ0402	handrail	Shihezi bicycle	Shihezi	*Staphylococcus haemolyticus*	I	S	R	R	S	S	S	S	S	S	R
8	BJ1101	handrail	Hellobike	Beijing	*Staphylococcus hominis*	S	S	S	R	S	R	S	S	S	S	S
9	BJ1102	handrail	Hellobike	Beijing	*Staphylococcus hominis*	S	S	R	S	S	S	R	S	R	S	I
10	BJ4601	handrail	ofo bike	Beijing	*Staphylococcus hominis*	S	S	R	S	S	S	S	S	S	S	S
11	BJ4602	handrail	ofo bike	Beijing	*Staphylococcus hominis*	S	S	R	S	S	S	S	S	S	S	S
12	JS08	handrail	Suzhou bicycle	Suzhou	*Staphylococcus hominis*	I	S	R	S	S	S	S	S	S	S	S
13	JS1001	handrail	Suzhou bicycle	Suzhou	*Staphylococcus hominis*	R	S	R	R	S	S	R	S	S	S	I
14	JS1002	handrail	Suzhou bicycle	Suzhou	*Staphylococcus hominis*	I	S	R	R	S	R	S	S	S	S	I
15	JS2101	handrail	Suzhou bicycle	Suzhou	*Staphylococcus hominis*	S	S	R	S	S	S	S	S	S	S	S
16	JS2102	handrail	Suzhou bicycle	Suzhou	*Staphylococcus hominis*	S	S	R	S	S	S	S	S	S	S	S

17	JS2403	handrail	Suzhou bicycle	Suzhou	*Staphylococcus hominis*	R	S	R	R	S	S	R	I	S	S	I
18	JS3301	handrail	Suzhou bicycle	Suzhou	*Staphylococcus hominis*	I	S	R	S	S	S	R	S	S	S	S
19	JS40	handrail	Suzhou bicycle	Suzhou	*Staphylococcus hominis*	I	S	S	S	S	S	S	S	S	S	I
20	JS4301	handrail	Suzhou bicycle	Suzhou	*Staphylococcus hominis*	I	S	I	R	S	R	S	R	S	S	S
21	JS4301	handrail	Suzhou bicycle	Suzhou	*Staphylococcus hominis*	S	S	S	S	S	S	S	S	S	S	S
22	JS4303	handrail	Suzhou bicycle	Suzhou	*Staphylococcus hominis*	S	S	S	S	S	S	S	S	S	S	S
23	JS4304	handrail	Suzhou bicycle	Suzhou	*Staphylococcus hominis*	I	S	I	R	S	R	S	R	S	S	I
24	JS49	handrail	Suzhou bicycle	Suzhou	*Staphylococcus hominis*	I	S	R	S	S	R	S	S	S	S	S
25	JS5001	handrail	Suzhou bicycle	Suzhou	*Staphylococcus hominis*	I	S	R	R	S	S	S	S	S	S	S
26	JS5002	handrail	Suzhou bicycle	Suzhou	*Staphylococcus hominis*	S	S	S	S	S	S	S	S	S	S	S
27	NX0202	handrail	Hellobike	Yinchuan	*Staphylococcus hominis*	S	S	R	S	S	S	S	S	S	S	S
28	NX0701	handrail	Mobike	Yinchuan	*Staphylococcus hominis*	I	S	R	R	S	R	S	S	S	S	S
29	QH1401	handrail	Xining bicycle	Xining	*Staphylococcus hominis*	I	S	R	R	S	R	S	S	S	S	I
30	QH1402	handrail	Xining bicycle	Xining	*Staphylococcus hominis*	I	S	R	R	S	R	S	S	S	S	S
31	QH1403	handrail	Xining bicycle	Xining	*Staphylococcus hominis*	I	S	R	R	S	R	S	S	S	S	S
32	SC10	handrail	Mobike	Chengdu	*Staphylococcus hominis*	S	S	S	R	S	S	S	S	S	S	S

33	SC3001	handrail	Hellobike	Chengdu	*Staphylococcus hominis*	R	S	S	S	S	S	S	S	S	S	S
34	SC3002	handrail	Hellobike	Chengdu	*Staphylococcus hominis*	R	S	S	R	S	S	S	S	S	S	S
35	SC35	handrail	Mobike	Chengdu	*Staphylococcus hominis*	I	S	R	S	S	S	S	S	S	S	S
36	SX0903	handrail	Mobike	Shuozhou	*Staphylococcus hominis*	S	S	S	R	S	S	S	S	S	S	S
37	SX1401	handrail	Mobike	Shuozhou	*Staphylococcus hominis*	S	S	R	R	S	S	S	S	S	S	S
38	SX1501	handrail	Mobike	Shuozhou	*Staphylococcus hominis*	S	S	R	S	S	R	S	S	S	S	I
39	SX21	handrail	Hellobike	Shuozhou	*Staphylococcus hominis*	R	S	R	R	S	S	S	S	S	S	S
40	SX3301	handrail	Hellobike	Shuozhou	*Staphylococcus hominis*	I	S	R	R	S	R	S	S	S	S	S
41	XJ0101	handrail	Shihezi bicycle	Shihezi	*Staphylococcus hominis*	I	S	I	S	S	S	S	S	S	S	S
42	XJ1602	handrail	Shihezi bicycle	Shihezi	*Staphylococcus hominis*	S	S	I	R	S	R	S	S	S	S	I
43	XJ17	handrail	Shihezi bicycle	Shihezi	*Staphylococcus hominis*	S	S	S	R	S	R	S	S	I	S	I
44	XJ1801	handrail	Shihezi bicycle	Shihezi	*Staphylococcus hominis*	R	R	I	S	S	R	S	S	S	S	S
45	XJ1802	handrail	Shihezi bicycle	Shihezi	*Staphylococcus hominis*	S	S	I	S	S	S	S	S	S	S	S
46	XJ1803	handrail	Shihezi bicycle	Shihezi	*Staphylococcus hominis*	S	S	I	S	S	S	S	S	S	S	S
47	XJ1804	handrail	Shihezi bicycle	Shihezi	*Staphylococcus hominis*	I	S	I	S	S	R	S	I	S	S	S
48	XJ2102	handrail	Shihezi bicycle	Shihezi	*Staphylococcus hominis*	I	S	I	S	S	S	S	S	S	S	I

49	XJ2103	handrail	Shihezi bicycle	Shihezi	*Staphylococcus hominis*	I	S	S	S	S	S	S	S	S	S	S
50	XJ2201	handrail	Shihezi bicycle	Shihezi	*Staphylococcus hominis*	I	S	I	S	S	S	S	S	S	S	S
51	XJ2202	handrail	Shihezi bicycle	Shihezi	*Staphylococcus hominis*	I	S	I	S	S	S	R	I	S	S	I
52	XJ2203	handrail	Shihezi bicycle	Shihezi	*Staphylococcus hominis*	I	S	R	S	S	S	R	S	S	S	S
53	XJ2301	handrail	Shihezi bicycle	Shihezi	*Staphylococcus hominis*	I	S	I	S	S	S	S	S	S	S	S
54	XJ2302	handrail	Shihezi bicycle	Shihezi	*Staphylococcus hominis*	I	S	I	S	S	R	R	S	S	S	S
55	XJ2303	handrail	Shihezi bicycle	Shihezi	*Staphylococcus hominis*	S	S	S	S	S	S	S	S	S	S	S
56	XJ2402	handrail	Shihezi bicycle	Shihezi	*Staphylococcus hominis*	S	S	R	S	S	S	S	S	S	S	S
57	XJ2701	handrail	Shihezi bicycle	Shihezi	*Staphylococcus hominis*	S	S	I	S	S	S	R	S	S	S	S
58	XJ2702	handrail	Shihezi bicycle	Shihezi	*Staphylococcus hominis*	S	S	R	S	S	S	S	S	S	S	S
59	XJ2901	handrail	Shihezi bicycle	Shihezi	*Staphylococcus hominis*	S	S	S	S	S	S	S	S	S	S	S
60	XJ2902	handrail	Shihezi bicycle	Shihezi	*Staphylococcus hominis*	S	S	R	R	S	S	S	S	S	I	I
61	XJ3001	handrail	Shihezi bicycle	Shihezi	*Staphylococcus hominis*	S	S	I	S	S	R	S	S	S	S	S
62	XJ3002	handrail	Shihezi bicycle	Shihezi	*Staphylococcus hominis*	I	S	I	S	S	S	S	S	S	S	S
63	XJ3201	handrail	Shihezi bicycle	Shihezi	*Staphylococcus hominis*	S	S	I	R	S	R	S	S	S	S	I
64	XJ3202	handrail	Shihezi bicycle	Shihezi	*Staphylococcus hominis*	S	S	R	S	S	S	S	S	S	S	S

65	XJ3501	handrail	Shihezi bicycle	Shihezi	*Staphylococcus hominis*	S	S	S	S	S	S	S	S	S	S	S
66	XJ4001	handrail	Shihezi bicycle	Shihezi	*Staphylococcus hominis*	S	S	I	S	S	S	S	S	S	S	S
67	XJ4002	handrail	Shihezi bicycle	Shihezi	*Staphylococcus hominis*	S	S	S	S	S	S	S	S	S	S	S
68	XJ4301	handrail	Shihezi bicycle	Shihezi	*Staphylococcus hominis*	I	S	S	S	S	S	R	S	S	S	S
69	XJ4302	handrail	Shihezi bicycle	Shihezi	*Staphylococcus hominis*	I	S	R	S	S	S	S	S	S	S	I
70	XJ4303	handrail	Shihezi bicycle	Shihezi	*Staphylococcus hominis*	I	S	S	S	S	S	S	S	S	S	S
71	XJ4304	handrail	Shihezi bicycle	Shihezi	*Staphylococcus hominis*	I	S	S	S	S	S	S	S	S	S	S
72	XJ4503	handrail	Shihezi bicycle	Shihezi	*Staphylococcus hominis*	S	S	S	S	S	S	S	S	S	S	S
73	XJ4601	handrail	Shihezi bicycle	Shihezi	*Staphylococcus hominis*	S	S	S	S	S	S	S	S	S	S	S
74	XJ4602	handrail	Shihezi bicycle	Shihezi	*Staphylococcus hominis*	I	S	I	R	S	R	S	S	S	S	I
75	XJ4603	handrail	Shihezi bicycle	Shihezi	*Staphylococcus hominis*	I	S	I	S	S	S	S	S	S	S	I
76	XJ4604	handrail	Shihezi bicycle	Shihezi	*Staphylococcus hominis*	S	S	S	S	S	S	S	S	S	S	S
77	XJ4801	handrail	Shihezi bicycle	Shihezi	*Staphylococcus hominis*	S	S	I	S	S	S	S	S	S	S	S
78	XJ4802	handrail	Shihezi bicycle	Shihezi	*Staphylococcus hominis*	S	S	S	S	S	S	S	S	S	S	S
79	XJ5001	handrail	Shihezi bicycle	Shihezi	*Staphylococcus hominis*	I	S	R	S	S	S	S	S	S	S	S
80	XJ5002	handrail	Shihezi bicycle	Shihezi	*Staphylococcus hominis*	S	S	S	S	S	S	S	S	S	S	S

81	XJ5003	handrail	Shihezi bicycle	Shihezi	*Staphylococcus hominis*	I	S	I	S	S	S	R	I	S	S	R
82	XJ5004	handrail	Shihezi bicycle	Shihezi	*Staphylococcus hominis*	I	S	S	R	S	R	S	S	R	S	I
83	YN2602	handrail	Qingju bicycle	Kunming	*Staphylococcus hominis*	I	S	I	R	S	R	S	S	S	S	S
84	YN2603	handrail	Qingju bicycle	Kunming	*Staphylococcus hominis*	I	S	I	R	S	R	S	S	I	S	I
85	SC4104	handrail	Mobike	Chengdu	*Staphylococcus kloosii*	S	S	S	R	S	S	S	S	S	S	R
86	JS2802	handrail	Suzhou bicycle	Suzhou	*Staphylococcus pseudintermedius*	R	R	R	R	S	R	S	R	S	S	I
87	NX1701	handrail	Mobike	Yinchuan	*Staphylococcus saprophyticus*	I	S	I	R	S	R	S	I	S	S	R
88	NX1702	handrail	Mobike	Yinchuan	*Staphylococcus saprophyticus*	R	S	R	R	S	R	S	S	S	S	I
89	NX1703	handrail	Mobike	Yinchuan	*Staphylococcus saprophyticus*	S	S	I	S	S	R	S	S	S	S	I
90	JS3201	handrail	Zhuzhou bicycles	Suzhou	*Staphylococcus sciuri*	R	S	I	R	S	S	R	S	S	S	R
91	NX1802	handrail	Mobike	Yinchuan	*Staphylococcus succinus*	S	S	I	R	S	R	R	I	I	S	I
92	NX19	handrail	Mobike	Yinchuan	*Staphylococcus succinus*	I	S	I	R	S	S	R	R	S	S	R
93	QH17	handrail	Xining bicycle	Xining	*Staphylococcus succinus*	S	S	I	R	S	S	S	I	S	S	R
94	QH3804	handrail	Xining bicycle	Xining	*Staphylococcus succinus*	I	S	I	R	S	S	S	R	S	S	I
95	QH3806	handrail	Xining bicycle	Xining	*Staphylococcus succinus*	S	S	I	R	S	S	S	S	S	S	I
96	SX3503	handrail	Hellobike	Shuozhou	*Staphylococcus succinus*	S	S	I	R	S	S	S	I	S	S	I

97	SX3504	handrail	Hellobike	Shuozhou	*Staphylococcus succinus*	S	S	I	R	S	S	S	S	S	S	I
98	SX3505	handrail	Hellobike	Shuozhou	*Staphylococcus succinus*	S	S	I	R	S	S	S	S	S	S	I
99	SX3501	handrail	Mobike	Shuozhou	*Staphylococcus succinus*	S	S	I	R	S	S	S	S	S	S	S
100	SX3502	handrail	Mobike	Shuozhou	*Staphylococcus succinus*	S	S	I	R	S	S	S	S	S	S	I
101	YN4401	handrail	Mobike	Kunming	*Staphylococcus warneri*	I	S	I	R	S	S	S	S	S	S	S
102	JS3303	handrail	Suzhou bicycle	Suzhou	*Staphylococcus xylosus*	S	S	S	S	S	S	S	S	S	S	S
103	QH3803	handrail	Xining bicycle	Xining	*Staphylococcus xylosus*	S	S	S	S	S	S	S	S	S	S	S
104	QH3807	handrail	Xining bicycle	Xining	*Staphylococcus xylosus*	R	S	S	R	S	R	S	S	S	S	I
105	SC0202	handrail	Mobike	Chengdu	*Staphylococcus xylosus*	R	S	I	R	S	S	S	S	S	S	I
106	SC03	handrail	Hellobike	Chengdu	*Staphylococcus xylosus*	R	S	I	R	S	S	S	S	S	S	I
107	YN4601	handrail	Mobike	Kunming	*Staphylococcus xylosus*	I	S	I	R	S	R	S	S	I	I	I
